# Laparoscopic treatment of renal hydatid cyst

**DOI:** 10.4103/0972-9941.51317

**Published:** 2009

**Authors:** Suraj C Prabhudessai, Roy V Patankar, Anil Bradoo

**Affiliations:** Department of Gastrointestinal and Minimally Access Surgery at Joy Hospital, India

**Keywords:** Endocyst, hydatid cyst, hypertonic saline, laparoscopy, renal

## Abstract

A 30-year-old woman was treated successfully for renal hydatid cyst disease by using the Transperitoneal Laparoscopic Technique. The peritoneal cavity was protected with the use of betadine-soaked gauze pieces, to avoid spillage. Hypertonic saline was used as the scolicidal solution to sterilize the cyst. The endocyst was removed completely and retrieved in an endobag. There were no intraoperative or early postoperative complications. This appears to be only the second reported case of renal hydatid cyst disease treated with the help of laparoscopy.

## INTRODUCTION

Hydatid cyst is a significant health problem in the sheep rearing areas of the world, with certain areas of India showing higher incidence. The treatment is mainly surgical and with appropriate diagnosis and treatment, prognosis is good. With advances and increasing experience in laparoscopic surgery, many more attempts have been made to offer the advantage of such a procedure to these patients.[1]

## CASE REPORT

A 36-year-old woman presented to our hospital with left-sided abdominal pain. The patient had no significant past medical history. An abdominal examination revealed left flank fullness. Abdominal sonography and a CT scan of the abdomen revealed a large left (10 × 12 cm) upper pole hydatid cyst in the left kidney. The remaining blood tests and urine examination were normal. No serological test was done and a diagnosis of renal hydatid cyst was made from the typical radiological finding. The patient was given albendazole for three weeks, preoperatively.

The patient had undergone sonography guided PAIR (puncture, aspiration, injection, re-aspiration) via the retroperitoneal route, by percutaneous means, on two occasions. Thus we decided to use the transperitoneal route, with necessary precautions, to avoid peritoneal contamination. The patient was given general anesthesia and placed in a supine position, with the left side elevated at 60 degrees. Pneumoperitoneum was established using a veres needle and CO2 insufflation. The retroperitoneum was exposed by mobilizing the left colon. The cyst was demonstrated and exposed all around by blunt dissection. The cyst was then isolated on all sides by packing it off using betadine-soaked gauze pieces (10% povidon iodine), to avoid contamination of the peritoneal cavity even if spillage occurred. A trocar was inserted into the cyst under vision, with due care taken to avoid spillage. The contents of the cyst were aspirated using an aspiration needle, and hypertonic saline was instilled for 10 minutes. The scope was inserted into the cyst to confirm complete evacuation. This was followed by cystotomy, and the endocyst was removed completely. This was introduced into an endobag. Partial cystectomy was carried out using a harmonic scalpel. Complete hemostasis was achieved. Omentoplasty was performed. The drain was kept in the retroperitoneum. The procedure lasted for 90 minutes. There were no intraoperative or early postoperative complications. The patient was discharged on the third day. The histopathological examination confirmed the diagnosis of hydatid cyst.

## DISCUSSION

Cystic hydatidosis is a disease caused by Echinococcus Granulosus, humans being the intermediate hosts. The location of the cyst is mainly hepatic (75%) and pulmonary (15%). Only 10% of the cysts occur in other parts of the body. Renal hydatidosis is usually associated with other organ involvement, and isolated renal disease is extremely rare, accounting for only 2 – 4% of the confirmed cases of hydatid disease. Radiological studies have an important place in the preoperative diagnosis of renal hydatid disease. Treatment of the renal hydatid cyst is surgical. Parenchyma saving surgery is the mainstay of the treatment, considering the benign nature of the disease. With advances in minimally invasive surgery, its benefits can be offered to patients with renal hydatid cyst. Conservative surgical treatment occupies a predominant role, and resection of a prominent dome is sufficient. Re-expansion of renal parenchyma occurs in a majority of cases.[2,3] Minimally invasive surgery has been used for the successful treatment of hydatid cyst in the lung and liver, according to the literature.[1,4] A review of the literature demonstrates that this is the second reported case of treatment of hydatid cyst of the kidney by laparoscopy.[5] We report a case of the laparoscopic management of renal hydatid cyst via the transperitoneal route. We had to resort to such a technique because the patient had undergone multiple percutanenous alcohol injections (PAIR) in the past, making the retroperitoneal route inaccessible. We performed cystotomy, with germinal membrane removal, after controlled evacuation and opening of the cyst. Intraperitoneal contamination was avoided by the use of a betadine-soaked gauze packed around the kidney. Contamination of the peritoneal cavity was further prevented by extraction of the germinal membrane in an endobag. Hence we conclude by stating that laparoscopic renal hydatid cyst surgery may be performed with minimal morbidity, minimal intraoperative blood loss, improved cosmetic results, shorter hospital stay, and an early return to work, making it the gold standard in the treatment of renal hydatid disease.
